# Smoothed Shock Filtering: Algorithm and Applications

**DOI:** 10.3390/jimaging7030056

**Published:** 2021-03-15

**Authors:** Antoine Vacavant

**Affiliations:** Institut Pascal, Université Clermont Auvergne, CNRS, SIGMA Clermont, F-63000 Clermont-Ferrand, France; antoine.vacavant@uca.fr

**Keywords:** image denoising, image enhancement, shock filtering, morphological operators, image segmentation, classification, robustness

## Abstract

This article presents the smoothed shock filter, which iteratively produces local segmentations in image’s inflection zones with smoothed morphological operators (dilations, erosions). Hence, it enhances contours by creating smoothed ruptures, while preserving homogeneous regions. After describing the algorithm, we show that it is a robust approach for denoising, compared to related works. Then, we expose how we exploited this filter as a pre-processing step in different image analysis tasks (medical image segmentation, fMRI, and texture classification). By means of its ability to enhance important patterns in images, the smoothed shock filter has a real positive impact upon such applications, for which we would like to explore it more in the future.

## 1. Introduction

Image enhancement and denoising consists of improving digital images by reducing inherent noise, which has been addressed by a wide variety of approaches [1,2,3,4]. Very popular or simple algorithms are generally employed for this task, such as Gaussian, median, or bilateral filterings [5,6,7], as they are implemented in many libraries dedicated to image processing (e.g., see OpenCV (http://opencv.org/—accessed on 12 March 2021), Matlab (http://mathworks.com—accessed on 12 March 2021) or ITK—Insight ToolKit (https://itk.org—accessed on 12 March 2021). The most recent advances deal with the development of deep neural networks [8,9,10], designed to learn input (Gaussian) noise and how to separate it from any new sample image.

Since the 1980s, numerous techniques based on PDE (Partial Differential Equations) have been proposed, starting with the well-known anisotropic diffusion introduced by Perona and Malik [11]. Other PDE-based approaches have been developed based on the concept of shock filtering, originally introduced by Kramer and Bruckner [12] and then popularized by Osher and Rudin [13]. Shock filters locally “shock” an image by erosion and dilation to create ruptures between local maxima and minima, by applying morphological operators depending on the sign of the Laplacian calculated on each pixel. This algorithm has several relevant theoretical properties: the range of output image’s values stays between the limits of the input image, contrary to other approaches such as Fourier transform- or wavelet-based ones; border effects such as Gibbs phenomenon cannot thus occur [14]; and it preserves the total variation of the processed signal and approximates deconvolution [15]. Another interesting property of this filter is its ability to enhance flow-like patterns, such as a fingerprints, a lion’s mane, or long hair. This principle is deeply investigated in [14,16], proposing the coherence-enhancing shock filter. Even if this PDE scheme is not originally able to process noisy signals, several authors have proposed extended versions [15,17,18].

In [18], we introduced an original extension of the shock filter by replacing standard morphological operators by smoothed dilation and erosion operators, following the work of Kass and Solomon [19]. The present article aims at describing this algorithm and the influence of parameters in Section 2. Then, we illustrate its application in diverse contexts. Firstly, we show how we measured its robustness against other similar PDE-based approaches for image denoising, by exploiting a formal definition of robustness in Section 3. Then, we present its positive impact as a pre-processing step in image segmentation and classification tasks in Section 4. Finally, we conclude the article and give future works in Section 5.

## 2. Smoothed Shock Filtering: Principle, Algorithm and Impact of Parameters

### 2.1. Algorithm Description

The original shock filter [13] processes each pixel p=(x,y) of an image *I* using the PDE scheme, given at iteration *t* by:

(1) $$I^{t}\left(p\right)=-\mathrm{sign}\left(\Delta I^{t-1}\left(p\right)\right)\left|\nabla I^{t-1}\left(p\right)\right|,\;t>0,$$

where $I^{0}\left(p\right)=I\left(p\right)$. $\Delta I^{t}\left(p\right)$ is the Laplacian operator calculated at p, ∇I is the gradient value at p. At each iteration t≥0 of this process, the filter applies morphological operators depending on the sign of $\Delta I^{t-1}\left(p\right)$:

(2) $$\left\{\begin{array}{c} \Delta I^{t-1}\left(p\right)<0\Rightarrow I^{t}\left(p\right)=I^{t-1}\left(p\right)⊕D\;\\ \Delta I^{t-1}\left(p\right)>0\Rightarrow I^{t}\left(p\right)=I^{t-1}\left(p\right)⊖D, \end{array}\right.$$

where *D* is a structural element of 1-pixel width and ⊕ and ⊖ are standard dilation and erosion operators. The shock filter employs these at local maxima and minima for each iteration, thus creating inflexion zones.

In [18], we proposed to improve this PDE scheme by integrating smoothed morphological operators inspired by the work of Kass and Solomon [19]. Those operators are defined by *smoothed local histograms* that are formalized as:

(3) $${\hat{f}}_{p}\left(s_{k}\right)=\sum_{p^{\prime }\in V\left(p\right)}K\left(I\left(p^{\prime }\right)-s_{k}\right)W\left(||p-p^{\prime }|{|}_{2}\right),\;1\leq k\leq n_{b},$$

where $n_{b}$ is the number of histogram bins, $s_{k}$ is the *k*th bin of this histogram, V(p) is the spatial neighborhood of p, and K,W are generally Gaussian kernels. They affect the impact of neighborhood on the current pixel in terms of intensity (*K*) and of position (*W*).

From this smoothed histogram, we obtain the integral by computing:

(4) $$R_{k}\left(p\right)=1-\left(C(I\left(.\right)-s_{k})*W\right)\left(p\right),\;1\leq k\leq n_{b}.$$

In this equation, *C* is the integral of *K*, expressed as an ERF (error function), and * is a standard convolution operator. To obtain a smoothed median filter, the algorithm consists of finding the $s_{k}$ value s.t. $R_{k}\left(p\right)=t$, with $t=\frac{1}{2}$. We can even come back to a standard median filter by defining the Gaussian kernel *W* with a std. equal to 0. Moreover, with this formalism, we can compute a smoothed dilation if we choose $\frac{1}{2}<t\leq 1$, and a smoothed erosion with $0\leq t<\frac{1}{2}$.

In our contribution, we employ the smoothed morphological operators in a shock filter scheme. In particular, we replace the standard dilation and erosion operators (see Equation (2)) by the calculation of the bin $s_{k}$ s.t.:

(5) $$R_{k}\left(p\right)=\left(\frac{1}{2}+\rho \Delta I\left(p\right)\right),$$

where ΔI(p)∈[−1;1] and $\rho \in [-\frac{1}{2};\frac{1}{2}]$ is a parameter fixed by the user. This equation means that we compute a smoother erosion of parameter $t=\frac{1}{2}-\rho$ when the Laplacian is positive (Equation (2)) and a smoothed dilation of parameter $t=\frac{1}{2}+\rho$ otherwise. Then, we impose that the processed pixel’s intensity $I^{\prime }\left(p\right)$ is equal to the value $R_{k}\left(p_{i}\right)$. By means of this process, we are able to process noisy signal with only a few iterations [7,18].

The complete sequence of instructions of the algorithm is exposed in Algorithm 1.
**Algorithm 1:** Smoothed shock filtering
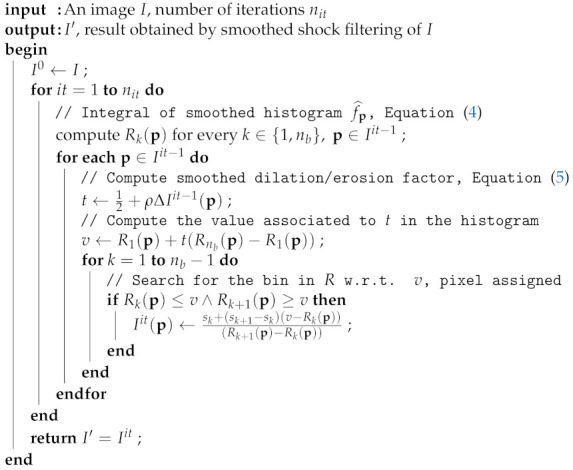


**Figure 1:**
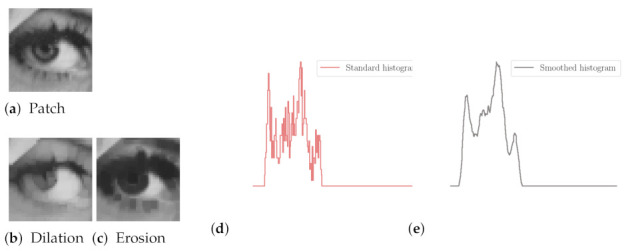
From a part of *Lena* image (**a**), i.e., V(p) around the central pixel p, the standard histogram (**d**) and smoothed histogram ${\hat{f}}_{p}$ from Equation (3) (**e**) are, respectively, depicted. We also present examples of smoothed dilation (**b**) and erosion (**c**) obtained over the whole patch.

For a given iteration of the filter $0\leq it\leq n_{it}$, the first step of this algorithm consists in calculating, for each pixel p of the input image, the smoothed local histogram over the $n_{b}$ bins, as depicted in Figure 1, by its integral $R_{k}\left(p\right)$ obtained with Equation (4) (Line 4). We then determine the percentage of smoothed erosion or dilation, represented by the value *t* on Line 6, depending on the Laplacian operator calculated at p and on ρ value set by the user (Equation (5)). We remind that, if *t* equals to 50%, we apply a smoothed median filtering at p, if t<50% a smoothed erosion, and a smoothed dilation otherwise (see Figure 1 for an illustration of those two latest processes). Line 7 represents the computation of *v*, which is the value of the smoothed histogram associated to *t*. The last part of Algorithm 1 aims at determining in the histogram *R* the $s_{k}$ bin associated to the *v* value. The last condition (Line 9) permits to searching for the two bins $s_{k},s_{k+1}$ in *R* that bound this searched value, associated to *v*. Thanks to the operation on Line 10, we obtain an interpolated value between $s_{k}$ and $s_{k+1}$ corresponding to *v*.

### 2.2. Impact of the Parameters

In this section, we present the influence of the parameters upon our smoothed shock filter. For color images, we consider the H channel only (with respect to HSV colorspace).

**Figure 2:**
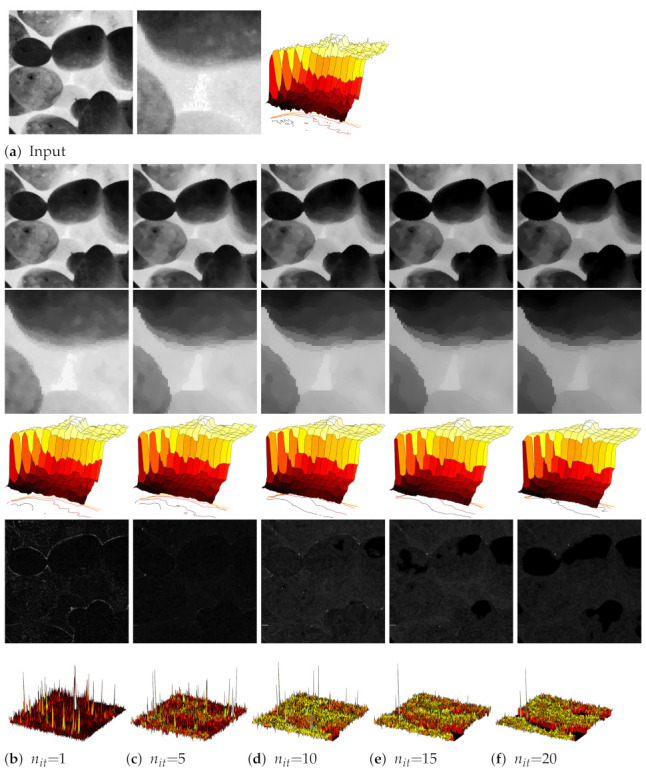
Scale-space representation obtained from input image (**a**), and the result from the filter for 20 iterations. The output images after 1, 5, 10, 15, and 20 iterations are presented, with a zoomed part and a height map. At the bottom, residual images are depicted with their corresponding height maps (**b**–**f**).

By increasing the number of iterations $n_{it}$, we obtain a *scale-space* representation of the input image, composed of the calculated images [20]. As an illustration, we depict in Figure 2 the successive outputs obtained from an image of the Brodatz dataset (http://www.ux.uis.no/~tranden/brodatz.html—accessed on 12 March 2021) [21], with $n_{it}=20$. We can appreciate the noise reduction, thanks to residual images and associated height maps, calculated from the difference between original and filtered images.

**Figure 3:**
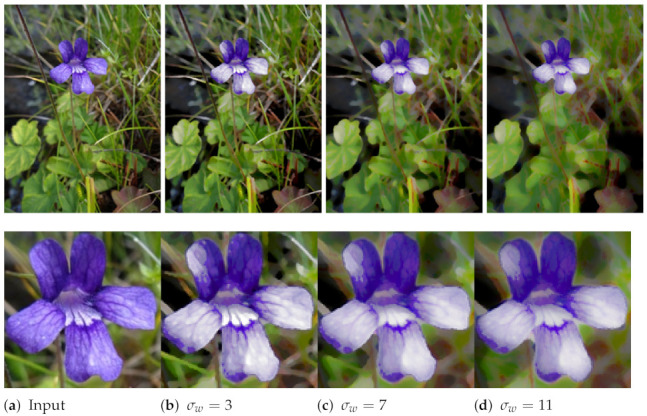
Impact of smoothing by augmenting the Gaussian kernel *W* with a flower picture (**top**) and a zoomed part of it (**bottom**).

Figure 3 presents a flower picture from the Tela Botanica database (http://www.tela-botanica.org/—accessed on 12 March 2021). We fixed here $n_{it}=20$, ρ=0.1, and changed the std. of the Gaussian kernel *W* (Equations (3) and (4)).

We can increase the size of the smoothing effect thanks to our filter in homogeneous regions (e.g., flower’s petals), while preserving contours. With high values of $\sigma_{w}$, the smoothed shock filter could serve as a water colorization technique.

**Figure 4:**
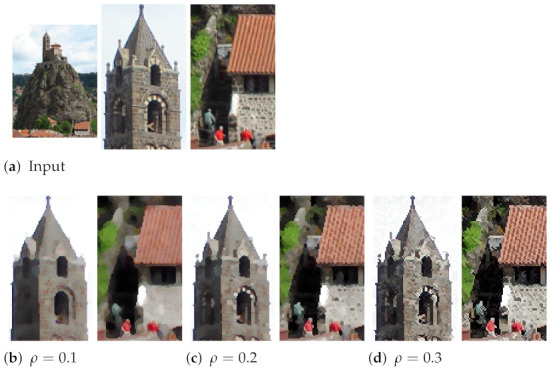
Impact of ρ parameter on smoothed morphological operators, with two zoomed parts of the image.

In our formalism, ρ adjusts the effect of the Laplacian operator upon the computation of smoothed morphological operators (Equation (5)). Figure 4 shows an image available under Creative Commons licence (https://commons.wikimedia.org/wiki/File:Rocher_St_Michel_%C3%A0_Aiguilhe.JPG—accessed on 12 March 2021). We set $n_{it}=20$ and an increasing ρ value, which decreases the smoothing effect. At the end, we obtained the standard shock filtering with ρ=0.5. In this experiment, we can notice that the algorithm preserves structural patterns including shingles and bricks even with small values of ρ.

Table 1 summarizes the parameters of our algorithm that can be set up for different applications.

## 3. A Robust Approach for Image Denoising

Whatever the application considered, a recurrent problem is the presence of an uncontrolled and destructive perturbation within image, coming from diverse sources: artifacts in medical acquisition machines, jittering videos from cameras due to wind, *etc.* This phenomenon comes from *data uncertainties*, such as *image noise*. The ability of an algorithm to resist to this noise (i.e., the algorithm’s output has been experimentally or theoretically guaranteed to be independent to noise) is commonly referred to *robustness* [7]. In [22], we proposed a first original and foundational definition of robustness dedicated to image processing algorithms, by getting inspiration from what has been introduced in computer vision. By considering multiple scales of additive (Gaussian) noise (with respect to standard deviation), we defined the α-robustness as the calculation of the worst quality loss (α) throughout the set of increasing noises. More recently, in [23], we introduced another quality-scale definition of robustness, which considers a more general notion of data uncertainties, and not only additive noise. As a consequence, we can consider more complex phenomena, in relation with image processing tasks. To evaluate this new measure of robustness, called (α,σ)-robustness, we still consider the α value presented above, together with the uncertainty scale σ for which the tested algorithm reaches its worst loss of quality.

This general definition can be described as follows. We consider an algorithm *A*, for a given image processing task, with an output $X={\left\{x_{i}\right\}}_{i=1,n}$ (generally the image directly obtained from *A*). Let *N* be a data uncertainty specific to the target application of *A*, and ${\left\{\sigma_{k}\right\}}_{k=1,m}$ the different scales of *N*. The outputs from *A* for each scale of *N* are denoted by $X={\left\{X_{k}\right\}}_{k=1,m}$. In addition, the ground truth is $Y^{0}={\left\{Y_{k}^{0}\right\}}_{k=1,m}$. Let now $Q(X_{k},Y_{k}^{0})$ be a quality measure of *A* for the scale *k* of *N*. The parameters of *Q* are the result of *A* and the ground truth for a noise scale *k*. An example can be the F-measure, combining true and false positive and negative detections for a binary decision (e.g., as binary segmentation). The definition of robustness that is employed hereafter can be expressed as:

**Definition** **1** ((α,σ)-robustness). *Algorithm A is considered robust if the difference between the output X and ground truth $Y^{0}$ is bounded by a Lipschitz continuity of the Q function:*

$$d_{Y}\left(Q\left(X_{k},Y_{k}^{0}\right),Q\left(X_{k+1},Y_{k+1}^{0}\right)\right)\leq \alpha d_{X}\left(\sigma_{k+1},\sigma_{k}\right),\;1\leq k<m,where$$

(6) $$\begin{array}{ccc} & & d_{Y}\left(Q\left(X_{k},Y_{k}^{0}\right),Q\left(X_{k+1},Y_{k+1}^{0}\right)\right)=Q\left(X_{k+1},Y_{k+1}^{0}\right)-Q\left(X_{k},Y_{k}^{0}\right),\\ & & d_{X}\left(\sigma_{k+1},\sigma_{k}\right)=\left|\sigma_{k+1}-\sigma_{k}\right|. \end{array}$$

*We calculate the robustness measure (α,σ) of A as the α value obtained and the scale $\sigma =\sigma_{k}$ where this value is reached.*

In this definition, α measures the worst drop in quality throughout data uncertainty scales $\left\{\sigma_{k}\right\}$ and σ represents the scale leading to this α value. As a consequence, the most robust algorithm should have both a low α value and a high σ value.

**Figure 5:**
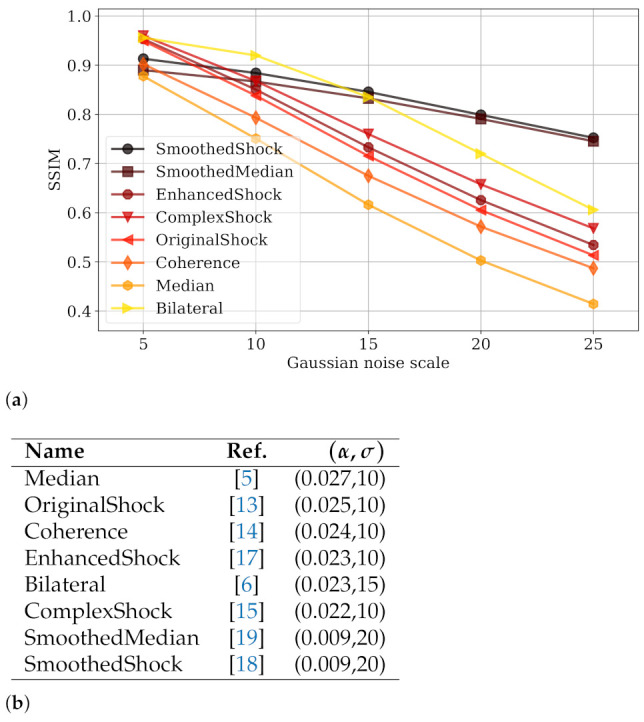
Evaluation of (α,σ)-robustness for several image denoising algorithms, compared to smoothed shock filtering, by studying quality function decrease through scales of noise (**a**) or numerically by appreciating the (α,σ) values for each algorithm (**b**).

In Figure 5b, we summarize the first approaches that we tested for image denoising. These are several approaches extending the shock scheme by regularization schemes [15,17] including ours [18]; the PDE-based coherence filtering discussed above [14]; the classic median [5] and bilateral [6] filterings; and the smoothed median filter introduced by Kass and Solomon [19].

In this experiment, we used the Denoiselab dataset [24], composed of 13 famous images (*Barbara*, *Airplane*, *etc.*), altered with additive white Gaussian noise with kernel std. ranging in the scales $\left\{\sigma_{k}\right\}=\left\{5,10,15,20,25\right\}$. This dataset thus contains 65 images of size 512 × 512 pixels. The quality measure is the SSIM (structural similarity) originally introduced by Wang [25].

By applying Definition 1, we measure the robustness of those algorithms (Figure 5), from a visual inspection with the graph in Figure 5a or by a quantitative approach by considering the (α,σ) values as in Figure 5b. As we consider an additive noise in this experiment, quality functions are decreasing monotonically over the set of noise scales. As a consequence, the tested algorithms progressively lose their efficiency throughout the scales of noise. We can observe that algorithms SmoothedMedian and SmoothedShock have a very good robustness, with both a lower α value and a larger σ scale than the other approaches. This means that the worst quality loss was observed when a more aggressive Gaussian noise is applied to images. The production of these plots and calculation of α and σ values can be reproduced thanks to a Python code diffused as a Github project (https://github.com/antoinevacavant/robustimageprocessing—accessed on 12 March 2021).

**Figure 6:**
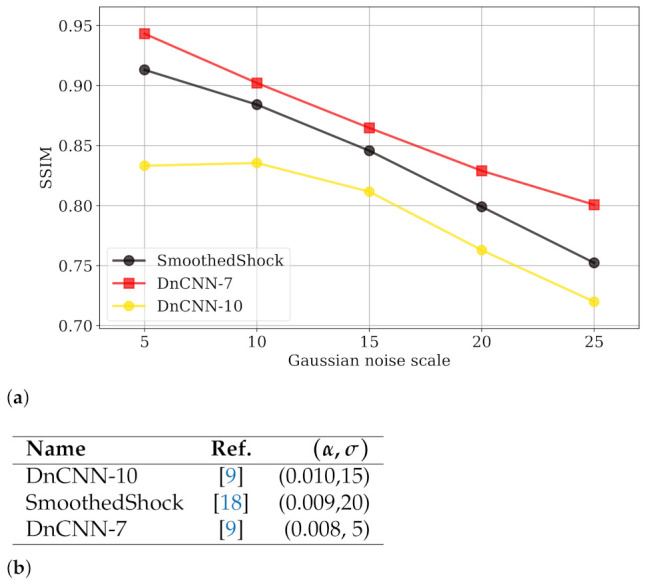
Evaluation of (α,σ)-robustness, by the graphical (**a**) and numerical (**b**) approaches, for the smoothed shock filtering and the DnCNN network, learned with two configurations.

We also numerically compared our method with the deep neural network DnCNN introduced by Zhang et al. [9]. This architecture is composed of convolutional layers and learns the residual noise from images. For the learning phase, we employed natural images from the BSDS500 dataset (https://www2.eecs.berkeley.edu/Research/Projects/CS/vision/bsds/—accessed on 12 March 2021), composed of 500 images, resized at 180 × 180 pixels, and then altered with additive white Gaussian noise with std. in the range {0,55} as the original article suggests. Then, we applied this model to denoise images from Denoiselab for testing phase. We used two setups: after 7 epochs (DnCNN-7) and after 10 epochs (DnCNN-10) of learning. The evaluation of robustness is depicted in Figure 6 (with a similar presentation as in Figure 5). With seven epochs, the SSIM of DnCNN-7 is higher than the one our SmoothedShock algorithm, for all noise scales of this test. The (α,σ)-robustness is also slightly better for this deep neural network. However, we can observe that a longer learning phase reduces the capability of generalization from the network; the global image quality and robustness of DnCNN suffer from this setup and are significantly lower than the ones of our approach.

**Figure 7:**
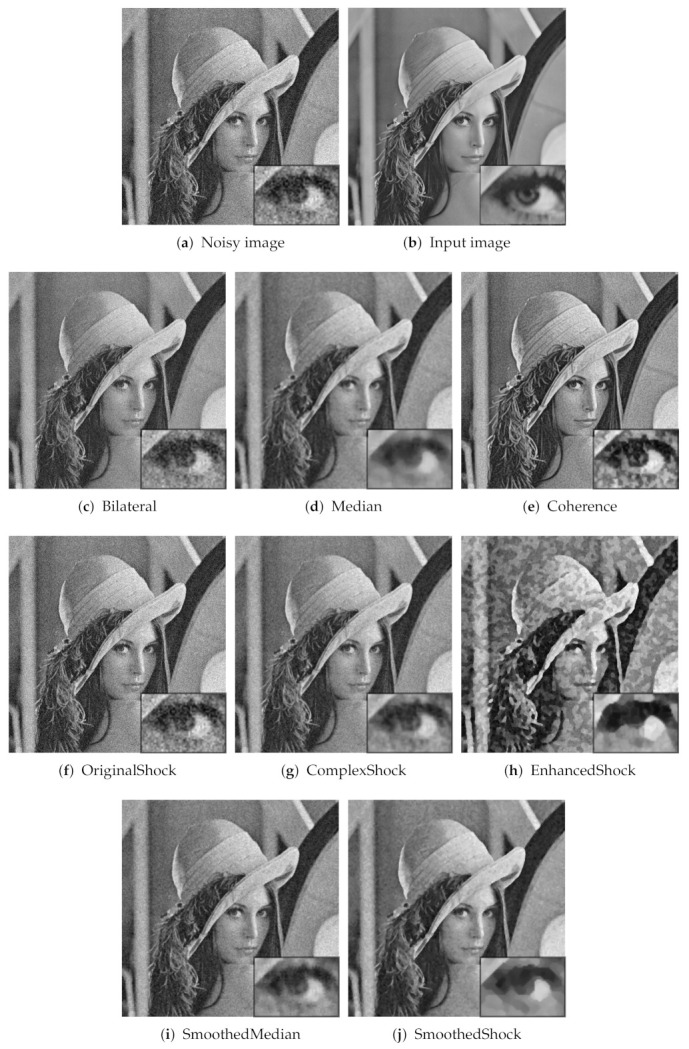
First illustrations of the results obtained for image denoising algorithms of our test.

In Figure 7, we present the outputs obtained for the first algorithms of our test. This figure confirms numerical results and shows the efficient image enhancement achieved by the most robust methods, SmoothedMedian and SmoothedShock.

**Figure 8:**
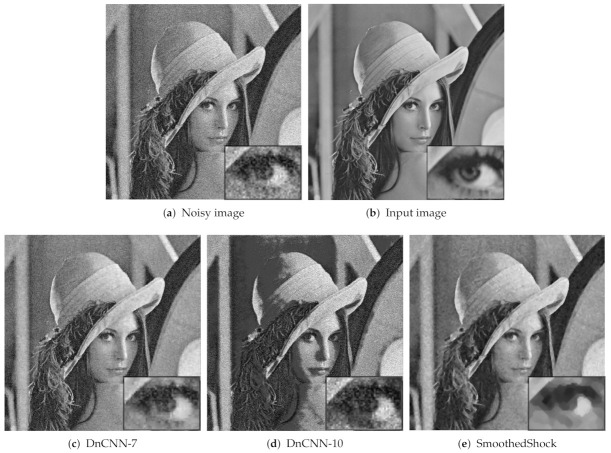
More illustrations of the results obtained for image denoising algorithms of our test.

Then, in Figure 8, we show the results obtained with the DnCNN approach, compared to ours, with the same image as in Figure 7. This illustration highlights the good quality of denoising from the DnCNN-7 model, which reduces noise from input image. Even if some noise is still present in the output image, geometries are preserved (e.g., the eye in the zoomed part), while our algorithm may modify boundaries by morphological operators. We can also observe that the DnCNN-10 alters dramatically image intensities, due to an inappropriate learning setup.

## 4. Image Enhancement for Improving Classification and Segmentation

We also exploited our algorithm in two main pattern recognition task: image segmentation and texture classification. In these projects, we mostly used the setup of parameters ρ=0.1 and $\sigma_{w}=3$ in our algorithm, while changing the number of iterations $n_{it}$.

### 4.1. Image Segmentation

**Figure 9:**
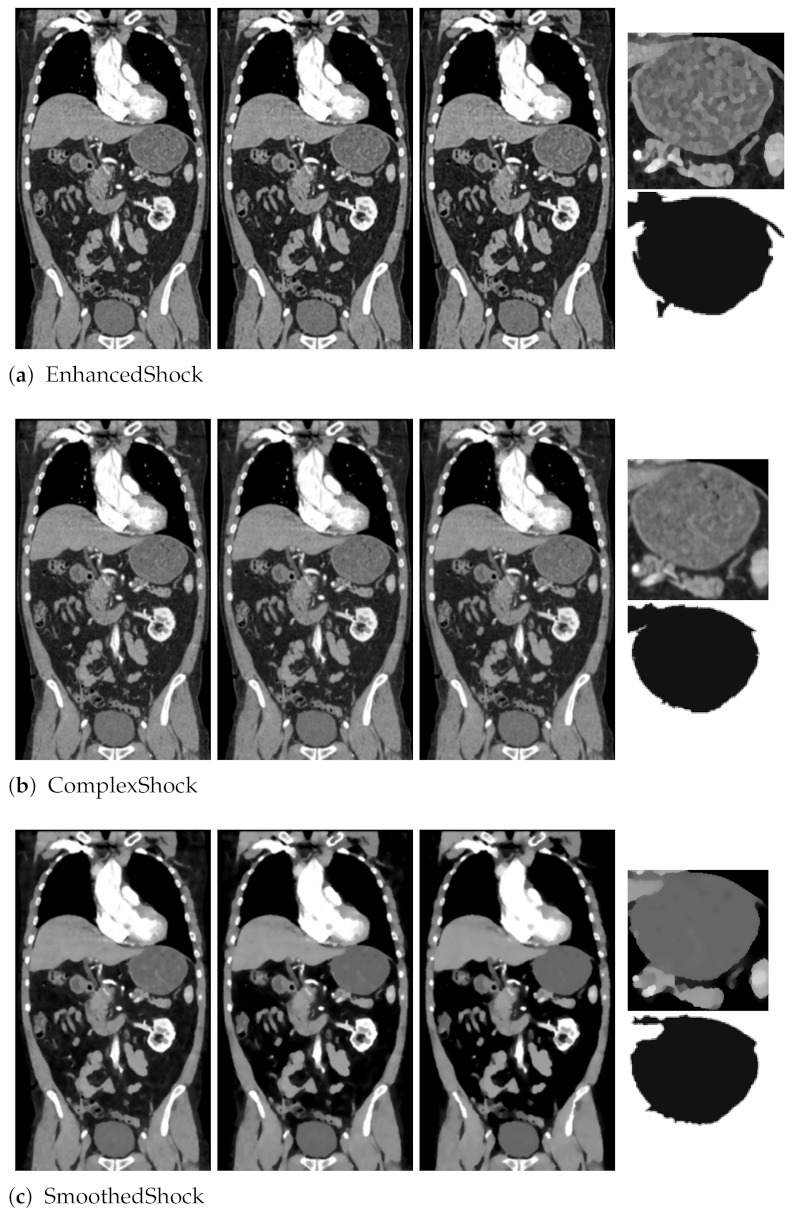
Segmentation obtained from one CT slice, with different methods, after 10, 20, and 30 iterations. A part of the segmentation is also presented.

As a preliminary test, we first proposed in [18] to exploit our filter as a pre-processing tool for medical image segmentation. As illustrated in Figure 9, we applied our filter and two others (EnhancedShock and ComplexShock) on a CT scan slice. After 10, 20, and 30 iterations, this evaluation revealed that our algorithm allows enhancing organs’ borders and internal tissues, which leads to an interesting segmentation, with a graph-based algorithm from Felzenszwalb and Huttenlocher [26] applied on images filtered 30 times.

**Figure 10:**
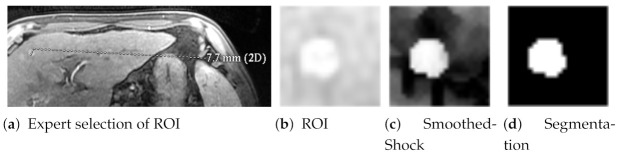
The steps of HCC segmentation from MRI slices.

In [27], we then proposed a complete pipeline devoted to the segmentation of liver cancer tissues in MRI slices. As exposed in Figure 10, it first consists of the extraction of regions of interest (ROI) within MRI images by a radiologist, in order to highlight the target cancerous nodule (HCC, hepatocellular carcinomas). This is a standard estimation of tumor’s viable tissues that may have higher intensities in MRI thanks to specific contrast agents. Smoothed shock filtering is the second step of our pipeline. Finally, we developed a fuzzification/defuzzification algorithm to segment the tumor.

This method first classifies image pixels into clusters by means of FCMVC (FCM with variable compactness) [28], an extension of classic FCM in which the size of the clusters is taken into account by a compactness parameter. As a defuzzification phase, we developed an original method inspired from Sequential Forward Floating Selection [29,30]. Its principle is to add pixels to the core, i.e. the highest non-empty α-cut in the fuzzy image, by considering geometrical and image intensity features (more details can be found in [27]).

**Figure 11:**
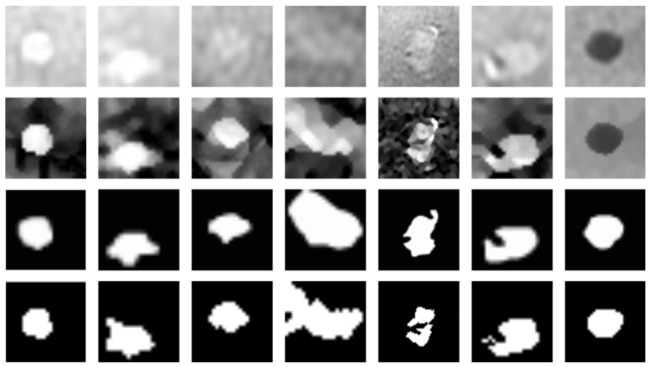
Segmentation results of HCC, from top to bottom: ROI selected by an expert; smoothed shock filter applied on these images; ground-truth of tumors’ contours; and segmentations obtained with our pipeline.

We present in Figure 11 the results obtained with our system for seven MRI ROI, representing different stages of HCC tumors: not treated and completely viable (first column), totally treated (last column), and others composed of partial necrosis. We can observe that the smoothed shock enhances the tissues and permits to extract efficiently the contours of the nodules. In [27], we also showed that our segmentation process is accurate for all these ROI, compared to other works selected from the literature.

### 4.2. Image Classification

In [7], we showed that our smoothed shock filter can be applied as an efficient pre-processing step for the classification of fMRI volumes. The application was to recognize brain activities from those images, and especially pleasure and disgust. In this context, fMRI scans were acquired while subjects were exposed to pleasant, neutral and disgusting pictures. Then, a classification system based on self-organizing maps was used [31,32] for the classification.

**Figure 12:**
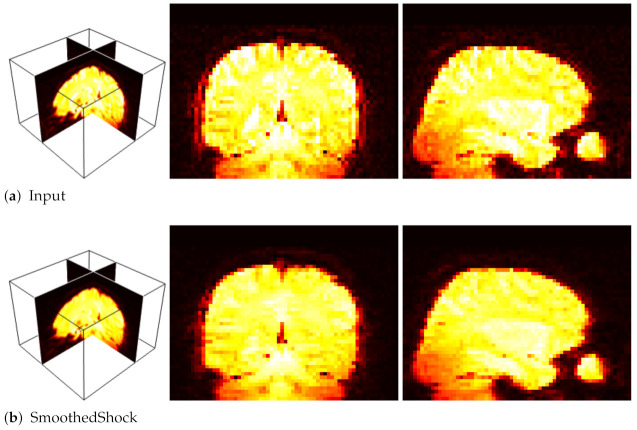
Filtering fMRI data: 3D view (**left**); and selected *X* and *Y* slices (**right**).

Our objective was to study the impact of smoothed shock filtering on the 3D images for a better recognition. To do so, we compared our filter with a standard fMRI pre-processing algorithm, FLIRT [33,34] and with related works. Table 2 exposes the numerical evaluation conducted with 10 fMRI sequences. Moreover, Figure 12 presents an example of fMRI volume processed with our filter, which smoothes homogeneous regions while preserving important patterns of brain activities.

We also used the smoothed shock filter to improve texture classification tasks in [35]. Firstly, we computed 20 new images from every texture belonging to a given dataset (one at each iteration of filtering). Then, filtered images and the original one were used as input for a feature descriptor selected from the literature: LBP (Local Binary Pattern), GLCM (Gray Level Co-occurrence Matrix), GLDM (Gray Level Difference Method), SFTA (Segmentation-based Fractal Texture Analysis), CLBP (Complete Local Binary Pattern), or LBPV (Local Binary Pattern Variance). Finally, a feature vector was computed as the concatenation of representations of filtered and original textures, obtained with our algorithm, compared to standard Gaussian filtering and anisotropic diffusion [11]. For a large-scale comparative study, we considered four datasets: Brodatz [21], composed of 111 textural classes with 10 samples each (the most used dataset for texture recognition in the literature and contains a variety set of images extracted from a photo album); Outex [36], with 68 classes with 20 images each (with 128×128 pixels, about natural scenes and surfaces, with intraclass variations such as viewpoint, scale and illumination); Vistex [37], with 864 images (the smallest dataset, presenting natural scenes with different scales, illuminations, viewpoints); and Usptex [38], composed of 2292 images, labeled in 191 classes (the largest dataset, containing different types of textures such as wood, plants, fabrics, among others).

Table 3 depicts the classification rates (with KNN and Naive Bayes) obtained with the three filters and without any pre-processing, together with the associated feature and iterations that permit to lead to these rates. We can observe that the smoothed shock enhances the texture at best for classification, for all the datasets. In most of the cases, local binary pattern and its variant CLBP are the most suitable textural descriptor.

## 5. Discussion and Future Works

In this article, we review the smoothed shock filter and its application in various image analysis tasks. Firstly, we explain the way to tune this algorithm in order to enhance and denoise differently input images. We then show that this is a robust and efficient approach for denoising and segmentation tasks improvement, thanks to its capability to smooth homogeneous regions, while preserving important contours. Finally, its use as a pre-processing step for image and texture classification is a real benefit.

There are several relevant advantages of using the smoothed shock filtering, compared to related works. First, we can choose setups in order to produce different outputs of the filter, for various applications (see Table 1). Thanks to the standard setup, we can achieve accurate enhancement for most applications exposed in this article, and certainly even more. By changing these parameters, we can also produce water colorization or sharpening effects for instance. Second, it is generally very difficult to determine which number of iterations should be set in most PDE-based algorithms, for achieving the best accuracy. Our algorithm only requires a few iterations (between two and five, as we showed in previous works [7,18]) to reach its best performance (in terms of, e.g., SSIM or PSNR). Third, our algorithm may be applied for denoising, but it is not its main purpose. As a consequence, it may be less efficient than a deep neural network such as DnCNN [9]. However, these end-to-end architectures are massively learned for denoising and not for enhancement as with ours. Furthermore, as observed in our experiments, their performance is highly dependent on the configuration of learning, and hyperparameterization, while smoothed shock filtering keeps a solid behavior with a simpler and faster tuning. Finally, by means of the smoothed morphological operators and the shock approach, we can enhance important patterns of images. This is an important feature for describing images’ content, as we did with the scale-space representation by combining iterations of the filter to best describe textures.

As future works, we first plan to compare our filter with more modern approaches based on other deep neural networks, according to standard metrics and robustness we define in this article. This comparison should take into account that those models require a very long training process (e.g., for DnCNN, more than 80 h for seven epochs and more than 110 h for 10 epochs with a standard PC), while our filter does not need any learning phase and can be tuned rapidly to obtain relevant results. In addition, we would like to exploit our filter in more contexts. As an example, we are working on its use as a pre-processing step for segmentation driven by a deep U-Net approach, so that we increase more network’s accuracy in biomedical image analysis tasks.

## Figures and Tables

**Table 1:** Different setups for smoothed shock filtering parameters.

Setup	Gaussian Kernel	Laplacian Operator	Number of Iterations
Standard, for most applications	$\sigma_{w}=3$	ρ=0.1	$1\leq n_{it}\leq 5$
Water colorization	$\sigma_{w}>3$	ρ=0.1	$n_{it}\geq 5$
Sharpening	–	ρ>0.1	$n_{it}\geq 5$
Scale-space representation	–	–	$n_{it}\geq 10$
Original shock filtering	–	ρ=0.5	–
Smoothed median filtering	$\sigma_{w}>0$	ρ=0.0	–
Median filtering	$\sigma_{w}=0$	ρ=0.0	–

**Table 2:** Performance of fMRI data classifier with different pre-processing filters.

Method	Recall	Precision	F-Measure
FLIRT	0.761	0.733	0.747
OriginalShock	0.797	0.777	0.787
Median	0.824	0.811	0.817
SmoothedShock	0.829	0.811	0.820

**Table 3:** Performance of classifiers, KNN (**a**) and Naive Bayes (**b**) approaches, for four texture classification datasets, by considering three pre-processing filters: smoothed shock filter, Gaussian filter, and anisotropic diffusion. For each filter, we present the best Correct Classification Rate (CCR), together with the feature and the combination of iterations leading to this rate. The leftmost column is the best rate without any pre-processing.

**(a) KNN**							
**Dataset**	**Original**	**Smoothed shock**		**Gaussian**		**Diffusion**	
	**CCR (feat.)**	**CCR (feat.)**	**It.**	**CCR (feat.)**	**It.**	**CCR (feat.)**	**It.**
Outex	75.59 (LBPV)	84.78 (GLDM)	{2,4,⋯,18}	83.01 (CLBP)	{9}	82.94 (CLBP)	{19}
Brodatz	97.6 (CLBP)	98.11 (CLBP)	{6}	97.20 (CLBP)	{2}	97.84 (CLBP)	{11,14}
Usptex	83.1 (CLBP)	88.66 (CLBP)	{1,8}	85.21 (CLBP)	{1}	88.57 (CLBP)	{1,3,⋯,17}
Vistex	98.96 (CLBP)	99.31 (CLBP)	{2}	98.96 (CLBP)	{2,3}	99.54 (CLBP)	{14}
**(b) Naive Bayes**							
**Dataset**	**Original**	**Smoothed shock**		**Gaussian**		**Diffusion**	
	**CCR (feat.)**	**CCR (feat.)**	**It.**	**CCR (feat.)**	**It.**	**CCR (feat.)**	**It.**
Outex	80.81 (LBP)	86.47 (LBP)	{2,7,⋯,17}	83.01 (LBP)	{8}	85.15 (CLBP)	{4}
Brodatz	96.6 (CLBP)	98.02 (CLBP)	{2}	96.85 (CLBP)	{1}	97.47 (CLBP)	{15}
Usptex	85.77 (CLBP)	91.49 (CLBP)	{1,3,5}	86.43 (CLBP)	{1,3}	89.66 (CLBP)	{8,11,⋯,20}
Vistex	97.33 (CLBP)	98.50 (CLBP)	{2,3}	98.50 (CLBP)	{1,3,5}	97.92 (CLBP)	{1,8}

## Data Availability

Not applicable.
